# Assessment of left ventricular function in patients with type 2 diabetes mellitus by non-invasive myocardial work

**DOI:** 10.3389/fendo.2023.1241307

**Published:** 2023-09-05

**Authors:** Wenjia Cao, Yan Deng, Linyi Lv, Xuebing Liu, Anguo Luo, Lixue Yin, Zhaohuan Li

**Affiliations:** ^1^ Department of Cardiovascular Ultrasound and Non-invasive Cardiology, Sichuan Provincial People’s Hospital, School of Medicine, University of Electronic Science and Technology of China, Chengdu, China; ^2^ Ultrasound in Cardiac Electrophysiology and Biomechanics Key Laboratory of Sichuan Province, Sichuan Provincial People’s Hospital, School of Medicine, University of Electronic Science and Technology of China, Chengdu, China

**Keywords:** myocardial work, type 2 diabetes mellitus, left ventricle, function, non-invasive

## Abstract

**Background:**

Diabetes mellitus (DM) is a chronic disease that poses a serious risk of cardiovascular diseases. Therefore, early detection of impaired cardiac function with non-invasive myocardial imaging is critical for improving the prognosis of patients with DM.

**Purpose:**

This study aimed to assess the left ventricular (LV) function in patients with type 2 diabetes mellitus (T2DM) by non-invasive myocardial work technique.

**Materials and methods:**

In all, 67 patients with T2DM and 28 healthy controls were included and divided into a DM group and a control group. Two-dimensional dynamic images of apical three-chamber view, apical two-chamber view, and apical four-chamber view were collected from all subjects, consisting of at least three cardiac cycles. LV myocardial strain parameters, including global longitudinal strain (GLS) and peak strain dispersion (PSD), as well as myocardial work parameters, including global constructive work (GCW), global wasted work (GWW), global work index (GWI), and global work efficiency (GWE), were obtained and analyzed.

**Results:**

A total of 15 subjects were randomly selected to assess intra-observer and inter-observer consistency of myocardial work parameters and strain parameters, which showed excellent results (intra-class correlation coefficients: 0.856 - 0.983, *P*<0.001). Compared with the control group, the DM group showed significantly higher PSD (37.59 ± 17.18 ms *vs.* 27.72 ± 13.52 ms, *P*<0.05) and GWW (63.98 ± 43.63 mmHg% *vs.* 39.28 ± 25.67 mmHg%, *P*<0.05), and lower GWE (96.38 ± 2.02% *vs.* 97.72 ± 0.98%, *P*<0.001). Furthermore, the PSD was positively correlated with GWW (r = 0.565, *P*<0.001) and negatively correlated with GWE (r = -0.569, *P*<0.001).

**Conclusion:**

Uncoordinated LV myocardial strain, higher GWW, and lower GWE in patients with T2DM may serve as indicators for the early assessment of cardiac impairment in T2DM.

## Highlights

- Uncoordinated LV myocardial strain, higher GWW, and lower GWE may be considered indicators of the early assessment of cardiac impairment in T2DM.
- Uncoordinated LV myocardial strain might be one of the reasons for increased GWW and decreased GWE.

## Introduction

The GBD Diabetes Collaborators have recently reported that the global burden of diabetes remains a significant public health challenge. In 2021, it was estimated that 529 million individuals were living with diabetes worldwide, with a global age-standardized total diabetes prevalence of 6.1%. By the year 2050, 89 out of 204 countries and territories will have an age-standardized rate of diabetes prevalence greater than 10% (1). Patients with type 2 diabetes mellitus (T2DM) have an increased incidence of cardiovascular diseases and are at high risk of experiencing sudden cardiac death (2, 3). Cardiac function impairment can occur at an early stage, even though the clinical manifestations of heart failure may not be obvious at this stage (4).

Early detection of impaired cardiac function is of great value for early treatment and improved prognosis. Cardiac magnetic resonance (CMR) is considered a precise non-invasive technique available for assessing cardiac structure and function. However, it possesses lower temporal resolution than echocardiography (5). Moreover, due to its expensive cost, time-consuming nature, and susceptibility to respiratory and arrhythmic artifacts, the clinical application of CMR remains somewhat restricted. In contrast, echocardiography, including tissue Doppler imaging, speckle tracking imaging, and non-invasive myocardial work, offers the advantages of convenience, affordability, and lack of ionizing radiation. Echocardiography is widely utilized in clinical settings. Non-invasive myocardial work is a new technique for evaluating myocardial function that provides a quantitative assessment of left ventricle function (6, 7). To date, only a limited number of studies have applied non-invasive myocardial work to explore the changes in myocardial function in patients with T2DM. However, some problems remain unsolved. First, the potential confounding effects of other coexisting conditions, including but not limited to hyperlipidemia and hypertension, on cardiac function injury have not been adequately addressed. Second, conventional ultrasound parameters have not been comprehensively evaluated in comparative studies (8–10). Finally, the results of some studies are controversial. Wang (8) and Yan (9) have suggested that individuals with T2DM exhibit increased global wasted work (GWW) and decreased global work efficiency (GWE) compared to the general population. However, Wang et al. found no statistically significant differences in GWW and GWE between the two groups (10). In the present study, we used a non-invasive myocardial work technique to obtain myocardial work parameters and compared them with conventional ultrasound parameters after excluding potential confounding factors. The findings of this study may provide evidence for the early diagnosis of impaired cardiac function in patients with T2DM.

## Materials and methods

### Study population

The DM group in this study consisted of 67 patients with T2DM who received treatment at Sichuan Provincial People’s Hospital between November 2018 to February 2019. Patients were required to meet the World Health Organization (WHO) standards (11), have no history of heart disease, and have normal physical examination and electrocardiogram (ECG) results. The inclusion criteria were as follows: (1) the presence of classic symptoms of hyperglycemia, including thirst, polyuria, weight loss, and blurry vision, along with a random blood glucose value of 200 mg/dL (11.1 mmol/L) or higher; (2) absence of classic symptoms of hyperglycemia, but the presence of any of the following criteria: fasting plasma glucose (FPG) values ≥ 126 mg/dL (7.0 mmol/L) (fasting defined as no caloric intake for at least 8 hours), two-hour plasma glucose values of ≥ 200 mg/dL (11.1 mmol/L) during a 75 g oral glucose tolerance test, and a glycosylated hemoglobin A1c (HbA1c) value ≥ 6.5% (48 mmol/mol). The diagnosis of diabetes was confirmed on a subsequent day by repeating the same test for confirmation or through two different tests that were concordant with the diagnosis of diabetes (12). The exclusion criteria were as follows: patients with type 1 DM, coronary atherosclerotic heart disease, congenital heart disease, cerebral infarction, peripheral arterial occlusive disease, valvular stenosis, valvular moderate or severe regurgitation, clinical diagnosis of heart failure, as well as those patients whose ultrasound examination revealed a left ventricular ejection fraction (LVEF) < 50%, intracardiac thrombus, intracardiac tumor, familial hypercholesterolemia, atrial fibrillation and other severe arrhythmias, Takayasu arteritis and other autoimmune diseases, liver or renal dysfunction, and poor ultrasound imaging quality of the heart. The control group consisted of 28 healthy volunteers who had no history of physical and laboratory evidence of DM, hypertension, hypercholesterolemia, or cardiovascular, cerebrovascular, or peripheral vascular diseases. These volunteers were matched to the patients in the DM group based on age and sex. The exclusion criteria of healthy controls were the same as the DM group. The study protocol was approved by the ethics committee of our hospital, and written informed consent was obtained from all participants.

### Demographic characteristics

Data including age, sex, and body mass index (BMI) were collected from all participants. Additionally, serum levels of triglyceride (TG), total cholesterol (TC), low-density lipoprotein cholesterol (LDL-c), high-density lipoprotein cholesterol (HDL-c), FPG, and glycosylated HbA1c were measured at the clinical laboratory department of our hospital. Measurements of systolic blood pressure (SBP), diastolic blood pressure (DBP), and heart rate were obtained from enrolled patients and healthy controls at the time of the acquisition of the apical views.

### Traditional LV function examination

All participants were subjected to a left lateral position for echocardiographic examination, and electrodes were connected to record the ECG. A GE Vivid E9 ultrasound machine (GE Inc., Boston, USA), equipped with an M5S phased array probe (1.5-4.6 MHz) (GE Inc., Boston, USA) was used to scan the heart while participants held their breath. After all the images were collected directly by an associate chief physician, a chief physician evaluated the image quality and the standard of the section, and selected graphics were recognized by both doctors for follow-up analysis. When there was a dispute, another chief physician was asked to evaluate or collect images again.

Pulsed Doppler was used to measure peak early-diastolic mitral velocity (E) and peak late-diastolic mitral velocity (A), while tissue Doppler imaging was used to measure the peak early-diastolic velocity (e’) of the lateral and septal mitral annulus. The average e’ was then calculated as (lateral e’+ septal e’)/2, while the Tei index was calculated as (time interval between the end of wave a and the start of wave e in the next cardiac cycle - time interval between the start and end of wave s)/time interval between the start and end of wave s. The endocardial boundary was delineated at the end-systolic and end-diastolic periods of the apical two-chamber and four-chamber views. This allowed for the automatic calculation of left ventricular end-systolic volume (ESV), end-diastolic volume (EDV), stroke volume (SV), and ejection fraction (EF).

### LV myocardial strain and myocardial work parameters acquisition

The two-dimensional dynamic images (at least three cardiac cycles) of the apical three-chamber view, apical two-chamber view, and apical four-chamber view, and the pulsed-wave Doppler images of the aortic valve and mitral valve were imported into EchoPAC workstation (GE Inc., Boston, USA) for analysis. The opening and closing nodes of the aortic and mitral valves were recorded by analyzing the blood flow spectra. A software program was utilized in conjunction with electrocardiography to identify dynamic images of three cardiac sections. Additionally, the left ventricular intima was auto-traced and manually corrected. Furthermore, blood pressure data was collected from the subjects and analyzed using the non-invasive myocardial work mode. Finally, the myocardial strain and myocardial work parameters of the left ventricle were obtained, including global longitudinal strain (GLS), peak strain dispersion (PSD), global constructive work (GCW), global wasted work (GWW), global work index (GWI), and global work efficiency (GWE).

### Repeatability of measurements

In all, the images of 15 subjects were randomly selected, and their myocardial strain parameters and myocardial work parameters were independently measured by Li Z (12 years of work experience) and Cao W (1 year of work experience). Two weeks later, Li Z reanalyzed the images.

### Statistical analysis

Statistical analysis was conducted using SPSS 22.0 (IBM Corp., Armonk, NY, USA). The measurement data were expressed as mean ± standard deviation (SD). To compare the two groups, the Student’s t-test and Wilcoxon rank sum test were used based on the normality and homogeneity of the variance of the data. Categorical data were expressed as n (%), and the χ^2^ test was used for between-group comparisons. Bivariate correlation was assessed using Pearson correlation analysis. Inter-group and intra-group consistency tests were performed by intra-class correlation coefficients (ICCs). P < 0.05 was considered statistically significant.

## Results

A total of 67 patients were enrolled in the DM group, with an average age of 59.85 ± 12.17 years, including 38 men. The control group consisted of 28 subjects with an average age of 57.63 ± 12.72 years, including 12 men.

### Demographic characteristics

Compared with the control group, patients with T2DM had significantly higher serum levels of FPG (10.59 ± 4.96 mmol/L vs. 4.98 ± 0.60 mmol/L, P<0.001) and higher levels of HbA1c (9.70 ± 3.70% vs. 5.11 ± 0.68%, P<0.001) (**Table 1**). No significant differences were observed in other baseline demographic characteristics between the two groups (P>0.05) (**Table 1**).

**Table 1:** Demographic characteristics.

	DM group (n=67)	Control group (n=28)	P-value
Age, years	59.85 ± 12.17	57.63 ± 12.72	0.433
Male, n (%)	38 (56.7%)	12 (42.9%)	0.157
BMI, kg/m^2^	25.21 ± 3.49	23.75 ± 2.24	0.144
SBP, mm Hg	131 ± 19	123 ± 18	0.073
DBP, mm Hg	76 ± 12	74 ± 9	0.353
HR, bpm	76 ± 12	74 ± 14	0.389
TG, mmol/L	2.44 ± 2.70	1.35 ± 0.55	0.095
TC, mmol/L	4.64 ± 1.24	4.56 ± 0.92	0.803
LDL-C, mmol/L	2.56 ± 0.97	2.90 ± 0.71	0.185
HDL-C, mmol/L	1.12 ± 0.35	1.26 ± 0.24	0.121
FPG, mmol/L	10.59 ± 4.96	4.98 ± 0.60	0.000
HbA1c, %	9.70 ± 3.70	5.11 ± 0.68	0.000

Values are expressed as mean ± standard deviation or number (%).

BMI, body mass index; SBP, systolic blood pressure; DBP, diastolic blood pressure; HR, heart rate; TG, triglyceride; TC, total cholesterol; LDL-C, low-density lipoprotein cholesterol; HDL-C, high-density lipoprotein cholesterol; FPG, fasting plasma glucose; HbA1c, glycosylated hemoglobin A1c.

### Differences in conventional ultrasound parameters between the DM and the control groups

Compared with the control group, the DM group showed slower lateral e’ (9.11 ± 2.70 cm/s *vs.* 10.88 ± 3.99 cm/s, *P*<0.05) and septal e’ (7.35 ± 2.25 cm/s *vs.* 8.85 ± 2.70 cm/s, *P*<0.01) (**Table 2**). However, there were no significant differences in ESV, EDV, SV, EF, E/A, E/average e’, and Tei index between the two groups (P>0.05) (**Table 2**).

**Table 2:** Conventional LV function parameters.

	DM group (n=67)	Control group (n=28)	P-value
E, cm/s	71.67 ± 17.10	68.52 ± 12.16	0.358
A, cm/s	78.90 ± 16.36	73.93 ± 18.88	0.236
E/A	0.94 ± 0.25	0.96 ± 0.22	0.621
Lateral e’, cm/s	9.11 ± 2.70	10.88 ± 3.99	0.046
Septal e’, cm/s	7.35 ± 2.25	8.85 ± 2.70	0.009
E/average e’	8.47 ± 2.52	8.01 ± 3.37	0.539
EDV, ml	70.69 ± 20.04	64.93 ± 17.07	0.200
ESV, ml	22.79 ± 8.30	21.56 ± 7.06	0.505
SV, ml	47.93 ± 13.97	43.41 ± 12.07	0.151
EF, %	68.04 ± 6.76	66.79 ± 6.53	0.426
Tei index	0.52 ± 0.16	0.49 ± 0.76	0.315

Values are expressed as mean ± standard deviation.

E, peak early-diastolic mitral velocity; A, peak late-diastolic mitral velocity; e’, mitral annular peak early-diastolic velocity; EDV, end-diastolic volume; ESV, end-systolic volume; SV, stroke volume; EF, ejection fraction.

### Differences in strain parameters and myocardial work parameters between the DM and the control groups

**Figure 1:**
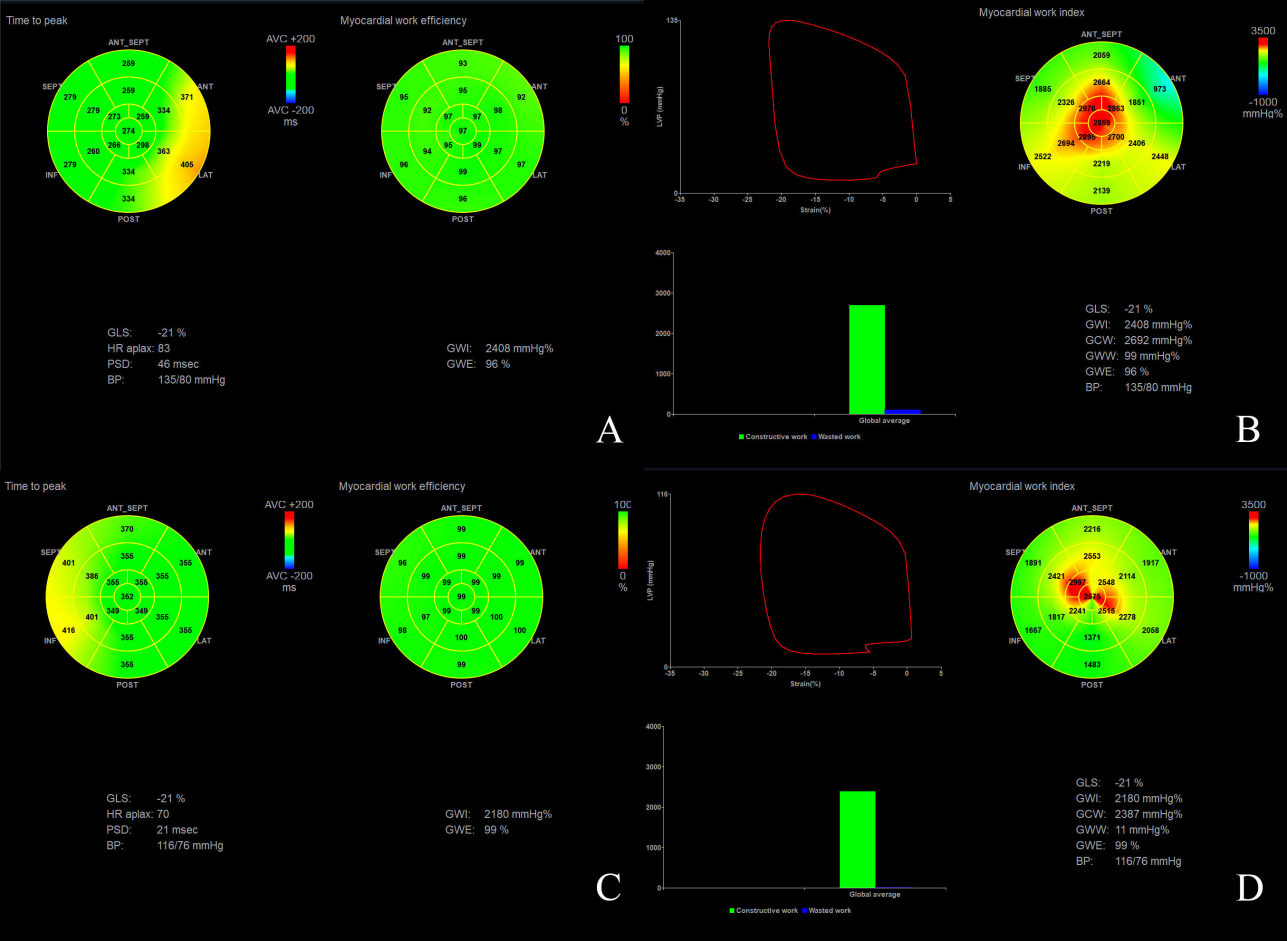
Myocardial work assessment of a patient with DM **(A, B)**, and a healthy subject **(C, D)**.

Although there was no statistically significant difference in GLS between the two groups, the proportion of patients with GLS > -20% was significantly higher in the DM group than in the control group (36 (53.7%) *vs.* 8 (28.6%), respectively, P<0.05) (**Table 3**). Furthermore, compared with the control group, the DM group exhibited increased PSD (PSD: 37.59 ± 17.18 ms *vs.* 27.72 ± 13.52 ms, P<0.05) and GWW (63.98 ± 43.63 mmHg% *vs.* 39.28 ± 25.67 mmHg%, P<0.05), and decreased GWE (96.38 ± 2.02% *vs.* 97.72 ± 0.98%, P<0.001, P<0.001) (**Figure 1**, **Table 3**). However, there was no significant difference in GWI and GCW between the two groups (P>0.05) (**Table 3**).

**Table 3:** Myocardial work parameters and strain parameters.

	DM group (n=67)	Control group (n=28)	P-value	
GLS, %	-18.94 ± 2.99	-19.68 ± 1.75	0.153
GLS > -20%, n (%)	36 (53.7%)	8 (28.6%)	0.025
PSD, ms	37.59 ± 17.18	27.72 ± 13.52	0.012
GWI, mmHg%	2026.35 ± 533.68	1959.36 ± 353.61	0.565
GCW, mmHg%	2297.16 ± 537.51	2259.52 ± 373.12	0.710
GWW, mmHg%	63.98 ± 43.63	39.28 ± 25.67	0.010
GWE, %	96.38 ± 2.02	97.72 ± 0.98	0.000

Values are expressed as mean ± standard deviation or number (%).

GLS, global longitudinal strain; PSD, peak strain dispersion; GWI, global work index; GCW, global constructive work; GWW, global wasted work; GWE, global constructive efficiency.

### Correlations of strain parameters and myocardial work parameters

**Figure 2:**
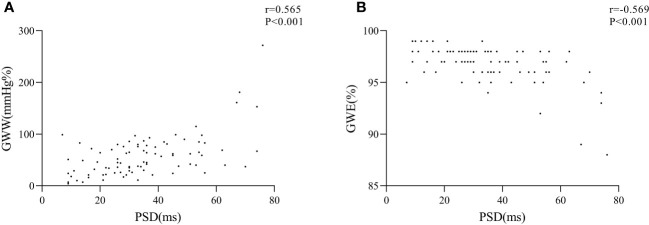
Correlation analysis of PSD and GWW **(A)** and GWE **(B)**.

The correlation analysis revealed a positive correlation between PSD and GWW (r = 0.565, P<0.001) and a negative correlation between PSD and GWE (r = -0.569, P<0.001) (**Figure 2**, **Table 4**).

**Table 4:** Pearson correlation analysis.

	PSD	
	r value	P-value
GWW	0.565	0.000
GWE	-0.569	0.000

PSD, peak strain dispersion; GWW, global wasted work; GWE, global constructive efficiency.

### Repeatability of measurements

The ICCs values of GLS, PSD, GWI, GWE, GCW, and GWW were all greater than 0.75 (P<0.001) (**Table 5**), indicating good reproducibility and reliability of the tests.

**Table 5:** Repeatability of measurements.

	ICC_1_(n=15)	P value	95%CI	ICC_2_(n=15)	P-value	95%CI
GLS	0.915	0.000	0.769-0.971	0.912	0.000	0.699-0.972
PSD	0.915	0.000	0.771-0.970	0.933	0.000	0.951-0.994
GWI	0.983	0.000	0.951-0.994	0.933	0.000	0.812-0.977
GCW	0.973	0.000	0.922-0.991	0.940	0.000	0.830-0.979
GWW	0.856	0.000	0.633-0.949	0.925	0.000	0.796-0.974
GWE	0.882	0.000	0.691-0.959	0.964	0.000	0.897-0.988

ICC_1_, Intra-group correlation coefficient; ICC_2_, Inter-group correlation coefficient. GLS, global longitudinal strain; PSD, peak strain dispersion; GWI, global work index; GCW, global constructive work; GWW, global wasted work; GWE, global constructive efficiency.

## Discussion

In 1972, Rubler and his colleagues first proposed the concept of diabetic cardiomyopathy, which suggested that DM is an independent causative factor of cardiovascular disease (13). Diabetic cardiomyopathy can cause myocardial hypertrophy and fibrosis due to metabolic disorders and other mechanisms, eventually leading to impaired diastolic and systolic functions. Cardiac diastolic dysfunction is more common and occurs earlier than systolic dysfunction (14–17). Echocardiography is a simple, non-invasive, and inexpensive method that can be used to diagnose heart failure, and evaluate prognosis and is considered the preferred imaging technique for clinical staff to evaluate myocardial function (18). Non-invasive myocardial work combines LV pressure with GLS to address the influence of afterload on LV deformation. This method allows for a more comprehensive and accurate evaluation of both local and global myocardial function (19–21). In this study, we found that a few LV diastolic function parameters were changed in patients with T2DM. Specifically, the lateral e’ and septal e’ were decreased, while the LV systolic function parameters, such as EF and Tei index, remained normal. Moreover, GWE was reduced, while PSD and GWW were increased in patients with T2DM compared with the controls. Therefore, PSD, GWW, and GWE may have potential value in evaluating early LV dysfunction in patients with T2DM.

### Assessment of LV diastolic function in patients with T2DM

To evaluate LV diastolic function, commonly used parameters include E/A, e’, E/average e’, etc. E and A reflect the pressure gradient between the left atrium and the LV in the early and late diastolic periods, respectively, and the ratio can be used to reflect the type of LV filling (including normal, abnormal, or pseudo-normalized) and evaluate LV diastolic function. The lateral e’ < 10cm/s and septal e’< 7cm/s are considered two indicators of abnormal LV diastolic function. E/average e’ can also be used to reflect LV filling pressure. E/average e’ < 8 usually indicates normal LV filling pressure, and E/average e’ > 14 is highly specific to increased LV filling pressure (14, 22). In this study, we found that compared with the control group, the amplitude of the motion of the mitral annulus in the DM group was reduced, suggesting that diastolic dysfunction may have existed in patients with T2DM.

### Evaluation parameters of LV systolic function in patients with T2DM

EF is the most used parameter for the clinical evaluation of LV systolic function. It reflects the proportion of blood ejected from the left ventricle into the systemic circulation during each cardiac cycle. Although GLS is less commonly used in clinical practice compared to EF, it is a more stable, repeatable, and suitable measure for evaluating subclinical cardiac dysfunction in patients (17). GLS reflects the shortening of the LV myocardium in the direction of the long axis during the systolic period, and a GLS value > -20% is suggested to indicate abnormal LV systolic function. A smaller absolute value indicates worse systolic function. PSD reflects the difference in time when the peak strain reaches each segment of the LV and is used to evaluate the synchronicity of LV myocardial deformation. In this study, the EF values of the two groups were similar and within the normal range. However, the PSD value and the number of patients with GLS>-20% were significantly higher in the DM group compared with the control group. This suggests that in patients with T2DM, LV myocardial deformation displays asynchrony and the longitudinal myocardial strain has been damaged to varying degrees, despite a normal EF.

### Evaluation of LV global function in patients with T2DM

LV non-invasive pressure-strain loop is generated through two-dimensional speckle tracking technology combined with the LV pressure curve, and it reflects the myocardial work (14). GWI reflects the total LV myocardial work from the mitral valve closure to its opening. GCW reflects the LV myocardial work done to facilitate ventricular ejection and relaxation by making myocardial shortening during systole and lengthening during diastole. Conversely, GWW reflects the LV myocardial work done to hinder ventricular ejection and relaxation by making myocardial lengthening during systole and shortening during diastole. GWE can be calculated as GCW/(GCW+GWW) (10, 23). The Tei index is the ratio of the sum of isovolumic contraction time and isovolumic relaxation time to the ejection time, and it reflects the overall systolic and diastolic function of the LV. An increase in the Tei index indicates a decrease in cardiac function. In this study, GWI, GCW, and Tei index were almost the same in the DM group compared to the control group, but GWW showed a significant increase and GWE was apparently reduced. In the early stage of diabetes, compensation may help maintain normal GWI, GCW, and Tei index. However, due to the increased PSD, some segments of the LV myocardium may reach the peak strain before the end of systole and begin to relax during systole, while some segments may not reach the peak strain at the end of systole, and continue to contract during diastole, leading to an increased GWW and a decreased GWE. Our study showed that PSD was positively correlated with GWW and negatively correlated with GWE, suggesting that uncoordinated LV myocardial strain might be one of the reasons for increased GWW and decreased GWE. Wang et al. found that LV GWW was increased, and the GWE was decreased in patients with T2DM, but their study showed a decrease in EF (5). In our study, the EF and Tei index of patients with T2DM was still normal, suggesting that the changes of LV GWW and GWE occurred earlier than changes in EF and Tei index, which would indicate that GWW and GWE might be more sensitive in detecting cardiac dysfunction in patients with T2DM.

### Limitations

We utilized a non-invasive cardiac ultrasound imaging technique to measure LV myocardial strain and myocardial work parameters. This method is safe, painless, and radiation-free. Moreover, we have provided a novel non-invasive technique for evaluating cardiac function in patients with T2DM. Our findings demonstrate that this technique can identify early signs of cardiac damage in these patients, providing important evidence and possibilities for early intervention and management. The present study has limitations that need to be acknowledged. First, the small sample size in this study limits the ability to perform deep subgroup analysis. Second, the potential influence of hypertension and dyslipidemia in patients with T2DM cannot be completely excluded. However, there was no statistically significant difference in the prevalence of hypertension and dyslipidemia between the two groups, suggesting that their influence might be relatively low. Finally, the history of medication use in T2DM patients was not discussed in this study.

## Conclusion

When only a few diastolic function parameters changed and EF and Tei index remain normal in patients with T2DM, PSD, GWW, and GWE seem to be more sensitive to subclinical LV dysfunction. Uncoordinated LV myocardial strain might be one of the reasons for the increased GWW and decreased GWE. Myocardial strain and myocardial work parameters have good reproducibility and may have potential value in detecting early LV dysfunction in patients with T2DM.

## Data availability statement

The raw data supporting the conclusions of this article will be made available by the authors, without undue reservation.

## Ethics statement

The studies involving humans were approved by the ethics committees of Sichuan Provincial People’s hospital. The studies were conducted in accordance with the local legislation and institutional requirements. Written informed consent for participation in this study was provided by the participants’ legal guardians/next of kin.

## Author contributions

ZL and LY designed the research. ZL and YD collected the ultrasound images. ZL, WC, and LL analyzed the ultrasound images. WC, ZL, and LY wrote the main manuscript text. XL and YD finished statistic analysis and prepared **Tables 1**–**5**. AL prepared **Figures 1** and **2**. All authors reviewed the manuscript. All authors contributed to the article and approved the submitted version.
